# Influence of Fiber Type and Length on Mechanical Properties of MICP-Treated Sand

**DOI:** 10.3390/ma15114017

**Published:** 2022-06-06

**Authors:** Shihua Liang, Xueli Xiao, Jie Wang, Yuxing Wang, Deluan Feng, Chengyuan Zhu

**Affiliations:** School of Civil and Transportation Engineering, Guangdong University of Technology, Guangzhou 510006, China; shihua_l@gdut.edu.cn (S.L.); 2112009005@mail2.gdut.edu.cn (X.X.); 2112109060@mail2.gdut.edu.cn (J.W.); 2112109097@mail2.gdut.edu.cn (Y.W.); 3119002913@mail2.gdut.edu.cn (C.Z.)

**Keywords:** MICP, length of fibers, mechanical properties, type of fibers, SEM

## Abstract

Fibers are applied in construction work to improve the strength and avoid brittle failure of soil. In this paper, we analyze the impact mechanism of fiber type and length on the immobilization of microorganisms from macroscopic and microscopic perspectives with fibers of 0.2% volume fraction added to microbial-induced calcite precipitation (MICP)-treated sand. Results show the following: (1) The unconfined compressive strength (UCS) of MICP-treated sand first increases and then decreases with increasing fiber length because short fiber reinforcement can promote the precipitation of calcium carbonate, and the network formed between the fibers limits the movement of sand particles and enhances the strength of the microbial solidified sand. However, the agglomeration caused by overlong fibers leads to uneven distribution of calcium carbonate and a reduction in strength. The optimal fiber length of polypropylene, glass, and polyvinyl alcohol fiber is 9 mm, and that of basalt fiber is 12 mm. (2) The UCS of the different fiber types, from small to large, is basalt fiber < polypropylene fiber < glass fiber < polyvinyl alcohol fiber because the quality of the fiber monofilament differs. More fibers result in more a evident effect of interlacing and bending on sand and higher strength in consolidated sand.

## 1. Introduction

Microbial-induced calcite precipitation (MICP) is an emerging soil reinforcement technology [1]. The technique refers to the process whereby specific microbial metabolites synthesize carbonates with substances in the surrounding environment [2]. The following chemical reactions are involved:

(1) $$\mathrm{CO}{\left({\mathrm{NH}}_{2}\right)}_{2}+2H_{2}O\to 2{\mathrm{NH}}_{4}^{+}+{\mathrm{CO}}_{3}^{2-}$$

(2) $${\mathrm{Ca}}^{2+}+{\mathrm{CO}}_{3}^{2-}\to {\mathrm{CaCO}}_{3}\left(s\right)$$

MICP has been used for many purposes, such as improvement of the physical and mechanical properties of soil [3], remediation of contaminated soil [4], repair of concrete cracks [5], restoration of ancient buildings [6,7], reinforcement of liquefied foundations [8], suppression of flying dust [9], prevention of rock and soil seepage [10], and mitigation of the greenhouse effect [11]. MICP is a popular research direction in geotechnical engineering, and the strength of sand reinforced by MICP is considerably improved, although shortcomings of brittle failure are observed [12,13,14,15], leading to the limitation of large-scale promotion and use of the technology.

**Figure 1:**
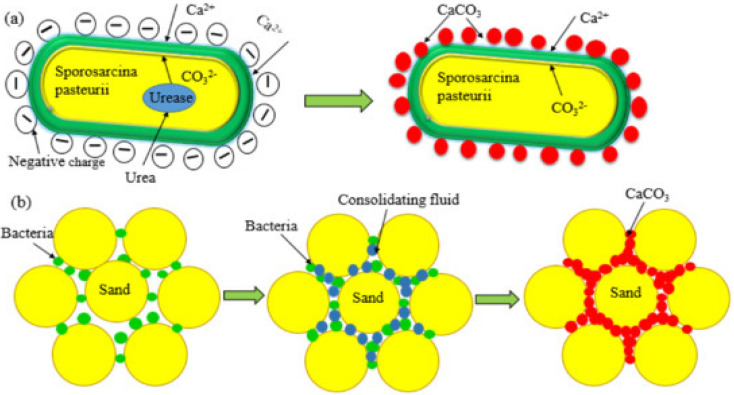
Schematic diagram of microbial solidified sand: (**a**) Microbial calcium carbonate production mechanism; (**b**) Calcium carbonate cemented sand granules.

In recent years, some studies have shown that the addition of discrete short-filament fibers to MICP-solidified soil can improve soil strength and effectively improve soil brittleness failure. Its mechanism is shown in Figure 1.

Mingdong Li et al. [16] found that the addition of an appropriate amount of fibers can remarkably improve unconfined compressive strength (UCS), ductility, and failure strain, and the optimal fiber content is 0.2–0.3%. Imran et al. [17] added fibers from Gastrodia elata during MICP curing to avoid the pollution caused by chemical fibers. The optimal fiber content and length are 3% and 15 mm, respectively. Choi S G et al. [18] added three different polyvinyl alcohol fiber contents to Ottawa sand in studying the microbial curing of sand and found that increased fiber content can improve UCS and split tensile strength and reduce brittleness. Zhao et al. [19] compared the mechanical behavior of four fiber-reinforced, MICP-cemented sands. However, different fiber types have dissimilar physical and mechanical parameters. When different types of fibers are incorporated into the MICP-cemented sand column, differences in the effect of fiber reinforcement are noted. In this paper, the effect of different fiber types and fiber lengths on the strength of microbial solidified sand is deeply analyzed, and the influence mechanism of strength is preliminarily explored.

In terms of economy, environmental protection, and reinforcement effect, after a large number of studies and comparisons [20,21,22,23], it has been found that polypropylene fibers, glass fibers, polyvinyl alcohol fibers, and basalt fibers are currently among the most frequently used fibers. Therefore, taking different fiber types (polypropylene fiber, glass fiber, polyvinyl alcohol fiber, and basalt fiber) and fiber lengths (3, 6, 9, and 12 mm) as the research objects for indoor tests and through comprehensive analysis of various physical and mechanical parameters, the influence mechanism of fiber length and fiber type on the strength of microbial solidified sand soil is explored.

## 2. Test Material

### 2.1. Experimental Bacteria

Sporosarcina pasteurii used in this study was purchased from DSM Company, the Netherlands, and numbered DSM33. A bacterial enlargement culture solution was prepared, and the components of the culture solution were 20 g/L yeast extract, 10 g/L ammonium sulfate, and 2 g/L sodium hydroxide. In each round, 50 mL of bacteria was used in each sample.

### 2.2. Experimental Fiber

**Figure 2:**
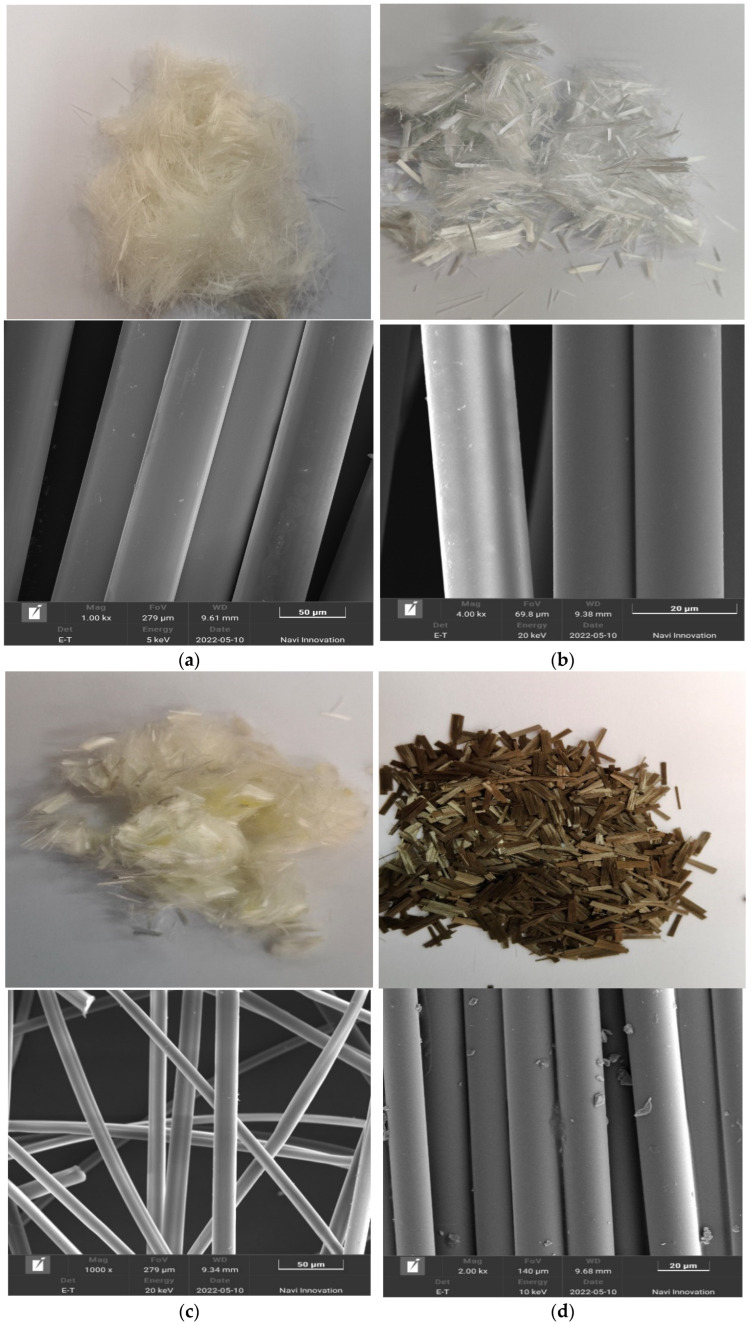
Physical and SEM micrographs of four fibers: (**a**) polypropylene fiber; (**b**) glass fiber; (**c**) polyvinyl alcohol fiber; (**d**) basalt fiber.

The fibers used in this test were polypropylene fiber, glass fiber, polyvinyl alcohol fiber, and basalt fiber. Their main physical and chemical properties are shown in Table 1 and Table 2, respectively. Physical morphology and SEM microscopic images of the four different types of fibers are shown in Figure 2. It is clear from the microscopic images that all four types of fibers have a relatively smooth surface; the polypropylene, glass, and polyvinyl alcohol fibers are in the form of dispersed monofilaments, whereas the basalt fibers are in the form of multiple monofilaments polymerized together in a flattened shape.

### 2.3. Sand for Test

**Figure 3:**
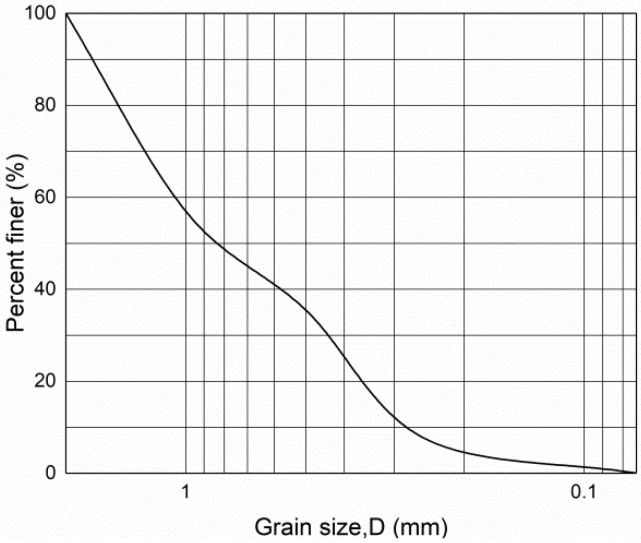
Grading size.

Fujian ISO standard sand was used in the test; the sand was characterized as fine, with uniform particles and poor gradation. Specific gravity of test sand: $G_{s}$ = 2.653,$\;D_{10}$ = 0.282,$\;D_{30}$ = 0.437, $D_{50\;}$ = 0.821, $\;D_{60\;}$ = 1.083. $D_{10}$ indicates the average particle size of sand with a mass fraction less than 10%. Unevenness factor: $C_{u}$ = 3.840. Curvature factor:$\;C_{c}\;$= 0.625, where the larger the inhomogeneity coefficient ($C_{u}$) the wider the grain group distribution of the sand. Figure 3 shows the specific particle gradation curve. 

### 2.4. Specimen Preparation

In this test, a PVC pipe with an inner diameter of 39.1 mm and a height of 115 mm was used to prepare the sample. The sample size was: diameter × height = 39.1 mm × 80 mm. Different fiber types (polypropylene fiber, glass fiber, polyvinyl alcohol fiber, and basalt fiber) and different fiber lengths (3 mm, 6 mm, 9 mm, and 12 mm) were mixed with microbial solidified sand with a fiber ratio of 0.2%; the sand and fibers were fully stirred and then compacted in three layers in the mold in 3 parallel samples [24,25].

### 2.5. Preparation of Nutrient Solution

In this test, the nutrient solution components were urea (30 g/L) and CaCl_2_ (55.50 g/L). The intermittent drip irrigation method was used. The bacterial solution and nutrient solution were injected into the sample by means of a peristaltic pump. The interval time of grouting was 8 h, with each round consisting of nine grouting applications and each sample injected with 50 mL of nutrient solution every round. Two rounds of grouting were carried out.

## 3. Experimental Test Methods

The treated sand samples were tested sequentially for the permeability coefficient test, water absorption test, dry density test, UCS test, calcium carbonate content test, and scanning electron microscopy (SEM) test.

### 3.1. Determination of Permeability Coefficient

The permeability test of variable water head was adopted for determination. The experimental steps were carried out according to the “Standards for Geotechnical Test Methods” 13.3.3 [26]. Put the cured sample into the permeation container, inject distilled water into the water head pipe to raise the water level to a certain height, cut off the water source after the water level is stable, open the water stop clip, and let the water pass through the sample when there is water in the water outlet. When it overflows, start recording the initial water head height and starting time in the water head pipe, wait a given time interval, and record the water level height in the water head pipe when the interval time ends. The permeability coefficient is calculated by Equation (3):

(3) $$k_{T}=2.3\frac{aL}{A\left(t_{2}-t_{1}\right)}log\frac{H_{1}}{H_{2}},$$

where a is the sectional area of the variable head pipe (${\mathrm{cm}}^{2}$); 2.3 is the transformation factor of ln and log; L is the seepage path, the height of the specimen (cm); $H_{1}$ is the starting head, and $H_{2}$ is the ending head (cm); and $t_{1}$ and $t_{2}$ are the start and end times of the reading head, respectively (s).

### 3.2. Determination of Water Absorption

First, the reinforced specimen was rinsed and soaked with deionized water and then put into the oven at 108 °C for drying. After it cooled down, its mass was weighed as $M_{1}$, and then the specimen was put into the saturated cylinder and soaked with deionized water for 24 h until it fully absorbed water. After completion, the specimen was taken out, and the residual water on its surface was quickly removed, and its mass was weighed as $M_{2}$. Water absorption is determined according to Equation (4):

(4) $$\omega =\frac{M_{2}-M_{1}}{M_{1}}\times 100\%$$

### 3.3. Determination of Dry Density

The reinforced specimens were placed in deionized water for soaking, then placed into an oven at 108 °C for drying, and the mass, height, and diameter of the specimens were measured after cooling. To ensure the accuracy of the measurement, the diameter of the specimen needs to be measured with vernier calipers on the top, middle, and bottom of the specimen, taking the average value. Dry density ($\rho_{d}$) is calculated according to Equation (5):

(5) $$\rho_{d}=\frac{M_{2}}{V},$$

where $M_{2}$ is the dry weight of the solidified soil sample (g), and V is the sand sample volume (${\mathrm{cm}}^{3}$).

### 3.4. UCS Test

For the unconfined compressive strength, a liquid crystal automatic pressure testing machine (YAW-S300) was used. A loading speed of 1.6 mm/min was used to load the sample to failure, and the ultimate load value at the time of failure was recorded.

### 3.5. Determination of Calcium Carbonate Content

Pickling was used for determination [27,28]. About 50 g of failure specimen of each group was taken, dried, called mass *M*_1_, and then placed in a beaker, adding excess hydrochloric acid to fully dissolve with internal calcium carbonate. After the reaction was complete, the pickled sand particle were rinsed with water several times and then called mass *M*_2_ after drying.

(6) $$W_{\left(C_{a}CO_{3}\right)}=\frac{M_{1}-M_{2}}{M_{1}}\times 100\%$$

### 3.6. SEM Test

A Zeiss Sigma 500/VP field emission SEM was used. A test block of appropriate size was placed in the vacuum chamber of the SEM after being sprayed with gold, and the typical shooting point was selected to zoom in 100, 200, 500, and 1000 times.

## 4. Results and Discussion

### 4.1. Physical Properties

From the physical and mechanical indicators of the sand column, it can be found that the sand samples with added polypropylene fibers, glass fibers, polyvinyl alcohol fibers, and basalt fibers had an increased amount of calcium carbonate precipitation in a certain range with increased fiber length. The amount of calcium carbonate produced in the sand samples using polypropylene fibers, glass fibers, polyvinyl alcohol fibers, and basalt fibers showed a peak or a slowed growth rate at a fiber length of 9 mm. The calcium carbonate content of the samples with 9 mm glass fibers was the highest, accounting for 150.6% of the unadded fiber; in general, the calcium carbonate content of the fiber-reinforced solidified sample was higher than that of the unaltered fiber sample, indicating that the added fiber is more conducive to the formation of calcium carbonate because the fiber is evenly distributed in the sand column. In the middle, the fibers filled in the gaps of the sand particles, which can increase the contact area of bacteria when perfusing the bacterial solution and provide more bacterial attachment points, resulting a large amount of calcium carbonate precipitates between the fibers and the sand particles. However, if the fiber length is too long, the fibers are prone to agglomeration in the sand column, making it difficult to mix evenly. The cemented fibers intercept a certain amount of bacteria, resulting in an uneven distribution of bacteria in the sand column, thereby affecting the formation of calcium carbonate location and generation.

**Figure 4:**
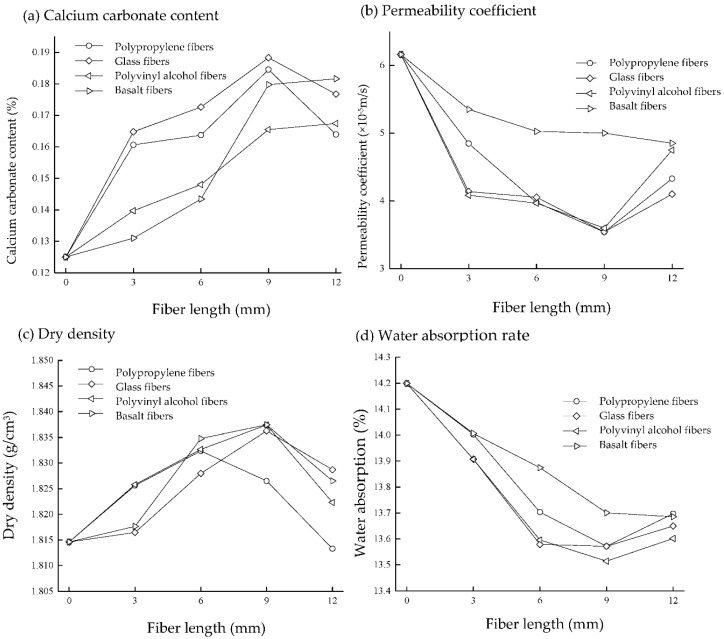
Physical properties: (**a**) Calcium carbonate content; (**b**) Permeability coefficient; (**c**) Dry density; (**d**) Water absorption rate.

Figure 4b shows that the permeability coefficient of the cured sand sample after the addition of fibers is 0.5 times smaller than that without added fibers. The reason is that whereas the fibers fill the pores of the sand particles, the precipitation of calcium carbonate is promoted so that the porosity of the sand particles is greatly reduced, thereby reducing the permeability coefficient. The data of dry density and water absorption show that the dry density of the MICP-cured sand sample mixed with the four fibers first increases and then decreases with increased fiber length, and the water absorption rate decreases first and then increases with increased fiber length. The amount of calcium carbonate precipitation is positively correlated with dry density and negatively correlated with the water absorption rate because the internal pores and volume of the sand column are fixed. The addition of fibers is beneficial to the formation of calcium carbonate precipitation. Fibers and calcium carbonate occupy the pores inside the sand sample, the connection between sand particles is tighter, the internal pore volume decreases, the water absorption capacity is weakened, and the quality of the sand column increases, specifically manifested as an increase in dry density and a decrease in water absorption.

### 4.2. Mechanical Properties

**Figure 5:**
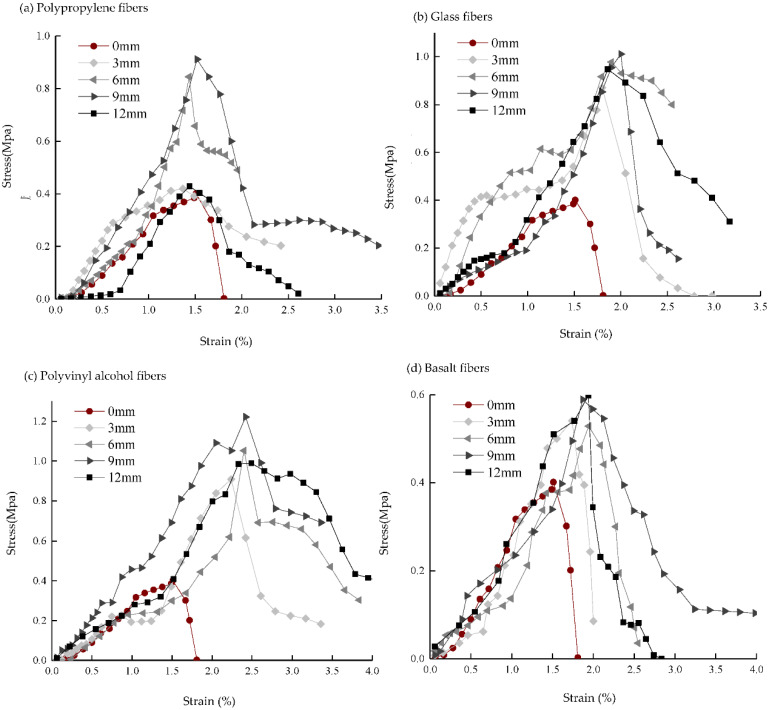
Stress–strain curve. (**a**) Polypropylene fibers; (**b**) Glass fibers; (**c**) Polyvinyl alcohol fibers; (**d**) Basalt fiber.

The stress–strain curves of sand columns with different types and lengths of fibers measured by the lateral limitless compressive test (Figure 5) show that under vertical loading, sand columns with different types and lengths of fibers result in an increase in stress with increasing strain at the initial stage until the peak and then a “stepwise” decline after the peak rather than a sharp decline. This is mainly because after the specimen is damaged, the fibers can cross the fissure like a “bridge,” bear a certain tensile stress, effectively inhibit the further development of the fissure, and delay the overall destruction of the specimen [28,29,30]. When the “stepped-tread platform” appears on the stress–strain curve, the addition of fibers can play a role in transmitting stress, preventing the expansion of cracks and the failure deformation of the sand column. The sharp drop after the “stepped tread” occurs because the fiber is pulled out or broken, the fiber can no longer transmit stress, and the crack is further expanded.

**Figure 6:**
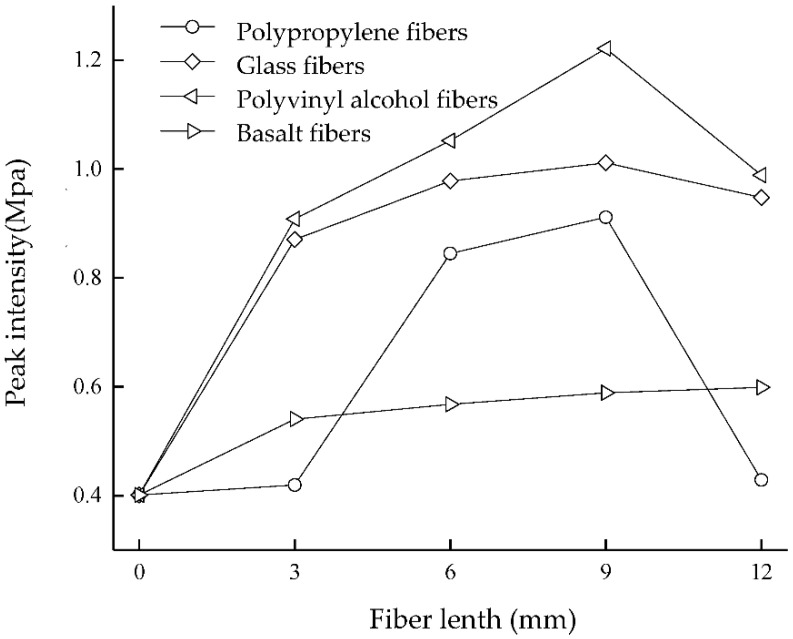
Unconfined compressive strength.

Figure 6 shows the peak of unconfined compressive strength. The addition of fibers can substantially increase the UCS of the MICP-cured sand. The UCS of the sand column increases with the length of the fibers within a certain length range. The strength of the sand column (with the addition of polypropylene fiber, glass fiber, and basalt fiber) reaches a peak at 9 mm, and the maximum strength can reach 0.912 MPa, 1.011 MPa, and 1.222 MPa, respectively. The overall parabolic characteristics are typical; the additional column strength with the addition of basalt fiber reaches a maximum value at 12 mm, with a value of 0.599 MPa. Comparing the effect of different lengths of fibers on microbially cured sandy soil, the optimal length of all three fibers occurs at 9 mm. The first reason is that the uniformly distributed fibers can effectively retain more bacteria in the sand column, which can generate more effective cemented calcium carbonate and improve the strength of the sand column [31]. The second reason is that the fiber also plays the role of a “bridge,” connecting the evenly distributed part of calcium carbonate with the unevenly distributed part [19,28]. The third reason is that when the sample is damaged and undergoes shear deformation, because the fiber strength is greater than the stress between sand particles, the fibers bear part of the tensile stress, limiting the deformation of the sample [32]. Thus, the fibers not only increase the strength of the sand column but also limit the movement of the sand particles. When the fiber length is too long, the fiber agglomeration phenomenon is evident [33,34]. The fibers in the sand column are not easy to mix evenly, resulting in an excess contact area between the fibers, reducing the contact area between the fibers and the sand particles. Thus, the bending effect of the fibers is weakened, so the strength of the sand column is reduced.

The peak strength measured by the unconfined compressive test reveals that the UCS of different fibers varies. The strength a achieve by the addition of fibers, from lowest to highest is: basalt fiber < polypropylene fiber < glass fiber < polyvinyl alcohol fiber. Table 1 shows that the monofilament diameter and quality of each fiber are as follows: polypropylene fiber > glass fiber > polyvinyl alcohol fiber. This means that the strength achieved with polyvinyl alcohol fiber is the highest, followed by glass fiber and polypropylene fiber. The more fiber monofilament added, the greater the frictional resistance between the sand and the soil, which increases the frictional resistance between the fiber and the sand, resulting in an improved pore filling effect in the sand. The stiffness of polyvinyl alcohol fiber monofilament is low, making it easy to bend and intertwine in the sand [35]. Therefore, the interweaving effect between fiber and sand particles is improved. More fiber roots are interwoven in the sand column to form a more solid three-dimensional network structure, which strengthens the spatial constraint of the fiber mesh structure on the soil body [36]. Although the monofilament quality of basalt fiber is inferior to that of glass fiber, during sample preparation, it is difficult to separate basalt fiber bundles into basalt monofilaments, and they tend to aggregate together in a flat shape [37]. Therefore, a certain number of basalt fiber bundles in the sand column is mixed with basalt fibers, and the 3D network structure formed in the sand column is poor.

### 4.3. Microstructural Features

**Figure 7:**
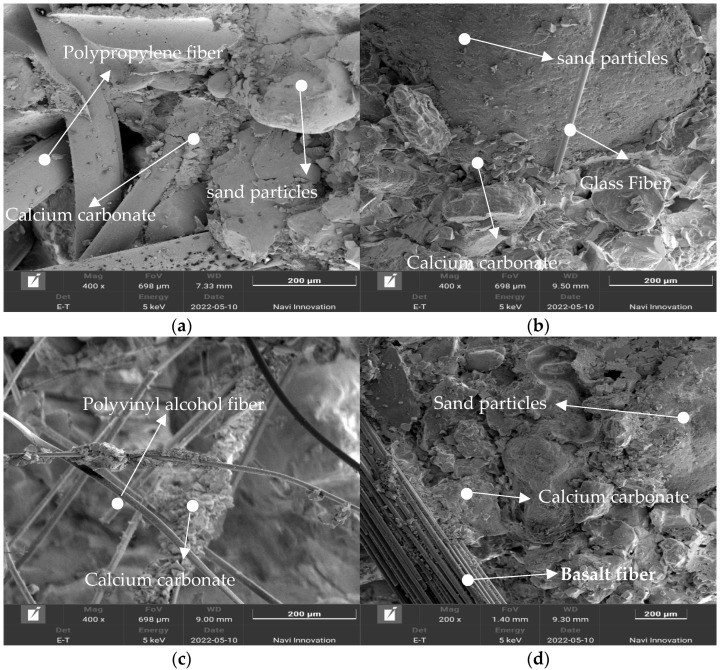
SEM images: (**a**) polypropylene fiber; (**b**) glass fiber; (**c**) polyvinyl alcohol fiber; (**d**) basalt fiber.

Figure 7 shows SEM images of four different kinds of fibers mixed into microbially cured sand; it is obvious that there is calcium carbonate precipitation at the location of contact between the fibers and sand particles. The generated calcium carbonate crystals have a multiprismatic block structure, the fibers are interspersed between the sand particles, and the calcium carbonate wraps the fibers and glues them together with the sand particles, which improves the roughness of the fiber surface and the occlusion force between the sand particles. Figure 7a,b shows that polypropylene fibers and glass fibers have a more obvious sand–fiber–calcium carbonate precipitation interface, and there are specimens destroyed when the fiber is pulled out of the groove. This indicates that in the process of destruction, in addition to the cementation of calcium carbonate and after a certain amount of tensile stress, the fibers will also bear a certain amount of tensile stress, “step tread” appears in the peak stress-strain curve, this occurs because the fibers in the fracture bear the tensile stress and inhibit the development of cracks. The appearance of a “step-kick surface” occurs precisely because the fibers are pulled out or pulled off, resulting in an inability to withstand the tensile force, resulting in further development of cracks, as well as a sudden reduction in axial stress.

The complex 3D mesh structure of polyvinyl alcohol fibers in Figure 7c, with a large number of fibers interwoven into a network, also proves that polyvinyl alcohol fibers have the most monofilaments at the same doping level and are more likely to bend to form a mesh structure. Calcium carbonate is deposited on the interweaving points between the fibers, which strengthens the restraining effect of the fiber network with the movement of sand particles. The fibers also play the role of “bridge” to connect the uniformly distributed portion of calcium carbonate with the unevenly distributed part so that the integrity of the sand column is enhanced.

In Figure 7d, the basalt fibers in the sand are generally in the form of fiber bundles, which are difficult to separate into monofilaments and cannot form a mesh structure that can bear stress. Therefore, the specimens with added basalt fibers have the lowest strength compared to other types of fibers.

## 5. Conclusions

(1) In microbial cured sand, reinforcement with short fibers has an effect of promoting the precipitation of calcium carbonate. The precipitation of calcium carbonate is negatively correlated with water absorption rate and positively correlated with dry density.
(2) Fiber length has an important effect on the mechanical properties of microbial cured sand soil. UCS increases first and then decreases with increased fiber length. When the length of polypropylene, glass, and polyethylene fibers is 9 mm and the length of basalt fibers is 12 mm, MICP has the best effect on sand samples.
(3) The effect of fiber type is greater than that of fiber length.
(4) The UCS of different fibers varies, and the strength, from lowest to highest, is basalt fiber < polypropylene fiber < glass fiber < polyvinyl alcohol fiber.

## Figures and Tables

**Table 1:** Physicomechanical behaviors of fibers.

Type	Density *ρ*/(g/cm^3^)	Line Density	Diameter	Tensile Strength/MPa	Elastic Modulus	Breaking Elongation	Breaking Strength
		/tex	/mm		/MPa	%	N/tex
polypropylene	0.91	——	0.025	≥500	≥3850	2.3	——
glass	2.699	4897	0.0174	2180	8720	2.52	——
polyvinyl alcohol	1.29	——	0.01509	1830	40,000	6.9	——
basalt	——	2392	——	3836	62,000	3.0	0.69

**Table 2:** Chemical composition of different fibers.

Fiber Type	Polypropylene Fiber	Glass Fiber	Polyvinyl Alcohol Fiber	Basalt Fiber
Chemical composition	${\mathrm{CH}}_{2}=\mathrm{CH}-{\mathrm{CH}}_{3}$ Polymers	${\mathrm{Al}}_{2}O_{3}$ , CaO , MgO $,\;{\mathrm{SiO}}_{2}$ $,\;{\mathrm{TiO}}_{2}$ $,\;{\mathrm{Na}}_{2}O$ $,\;B_{2}O_{3}$	${\left(C_{2}H_{4}O\right)}_{n}$	${\mathrm{Al}}_{2}O_{3}$ , CaO , MgO $,\;{\mathrm{SiO}}_{2}$ , FeO $,\;{\mathrm{TiO}}_{2}$

## Data Availability

Not applicable.
